# Catabolism of mucus components influences motility of *Vibrio cholerae* in the presence of environmental reservoirs

**DOI:** 10.1371/journal.pone.0201383

**Published:** 2018-07-26

**Authors:** Geethika Reddi, Kali Pruss, Kathryn L. Cottingham, Ronald K. Taylor, Salvador Almagro-Moreno

**Affiliations:** 1 Burnett School of Biomedical Sciences, College of Medicine, University of Central Florida, Orlando, Florida, United States of America; 2 Department of Microbiology & Immunology, Stanford School of Medicine, Stanford University, Palo Alto, California, United States of America; 3 Department of Biological Sciences, Dartmouth College, Hanover, New Hampshire, United States of America; 4 Department of Microbiology and Immunology, Geisel School of Medicine, Dartmouth College, Hanover, New Hampshire, United States of America; 5 National Center for Integrated Coastal Research, University of Central Florida, Orlando, Florida, United States of America; East Carolina University Brody School of Medicine, UNITED STATES

## Abstract

*Vibrio cholerae* O1, the etiological agent of cholera, is a natural inhabitant of aquatic ecosystems. Motility is a critical element for the colonization of both the human host and its environmental reservoirs. In this study, we investigated the molecular mechanisms underlying the chemotactic response of *V*. *cholerae* in the presence of some of its environmental reservoirs. We found that, from the several oligosaccharides found in mucin, two specifically triggered motility of *V*. *cholerae* O1: *N*-acetylneuraminic acid (Neu5Ac) and *N*-acetylglucosamine (GlcNAc). We determined that the compounds need to be internally catabolized in order to trigger motility of *V*. *cholerae*. Interestingly, the catabolism of Neu5Ac and GlcNAc converges and the production of one molecule common to both pathways, glucosamine-6-phosphate (GlcN-6P), is essential to induce motility in the presence of both compounds. Mutants unable to produce GlcN-6P show greatly reduced motility towards mucin. Furthermore, we determined that the production of GlcN-6P is necessary to induce motility of *V*. *cholerae* in the presence of some of its environmental reservoirs such as crustaceans or cyanobacteria, revealing a molecular link between the two distinct modes of the complex life cycle of *V*. *cholerae*. Finally, cross-species comparisons revealed varied chemotactic responses towards mucin, GlcNAc, and Neu5Ac for environmental (non-pathogenic) strains of *V*. *cholerae*, clinical and environmental isolates of the human pathogens *Vibrio vulnificus* and *Vibrio parahaemolyticus*, and fish and squid isolates of the symbiotic bacterium *Vibrio fischeri*. The data presented here suggest nuance in convergent strategies across species of the same bacterial family for motility towards suitable substrates for colonization.

## Introduction

Cholera is a severe diarrheal disease caused by the facultative pathogen *Vibrio cholerae* O1 [1,2]. The disease remains a major scourge in many developing countries, with estimates of 3 to 5 million people contracting cholera yearly, leading to 100,000 to 120,000 deaths [1,2]. Seven pandemics of cholera have been recorded to date. The first six pandemics of cholera were caused by strains belonging to the classical biotype [1,2]; the current pandemic is caused by strains of the El Tor biotype and has spread across the globe in several waves of transmission [3].

*V*. *cholerae* belongs to the family Vibrionaceae, a varied group of aquatic organisms that encompasses other human pathogens such as *Vibrio vulnificus* or *Vibrio parahaemolyticus*, as well as symbiotic species such as *Vibrio fischeri* [4,5]. *V*. *cholerae* O1 is a facultative pathogen and a natural inhabitant of aquatic environments, such as rivers, estuaries and oceans, where it can be found free-living or attached to biotic or abiotic surfaces [6–8]. *V*. *cholerae* O1 has been found associated with several inhabitants of its natural environment such as crustaceans, cyanobacteria or oysters [9–15]. Cholera is transmitted to humans by consumption of water or food contaminated with virulent strains of *V*. *cholerae* O1 [1,2]. After entering the host, the cells that survive the acidic environment of the stomach must penetrate a highly viscous layer of mucus covering the intestine in order to successfully attach to the epithelial cells of the small intestine, then proliferate to generate a productive infection [16,17]. Motility has been shown to be a crucial element for *V*. *cholerae* to colonize the epithelium and cause a successful infection of the human host [17–24]. Liu *et al* recently showed that mucin affects the motility of *V*. *cholerae* while repressing polysaccharide synthesis [25]. Intestinal adherence of the bacterium involves a coordinated interaction between mucin and GbpA, an adhesin that mediates attachment to intestinal epithelial cells and the chitinaceous surface of crustaceans [26,27]. In the aquatic environment, given the relative scarcity of nutrients, the bacterium needs to accurately recognize the presence of, and move towards, habitats with suitable nutrient conditions [9,28–31].

Both the human intestinal epithelium and some critical environmental reservoirs of *V*. *cholerae*, such as cyanobacteria or the gills of mollusks, are covered by a layer of mucus, which is comprised of large and viscous glycoproteins of high molecular weight called mucins. Mucins are large, with molecular weights ranging from 0.5 to 20 megadaltons. Membrane-bound and secreted mucins share many common features; for instance, they are both highly glycosylated and consist of 80% carbohydrates; primarily *N*-acetylgalactosamine (GalNAc), *N*-acetylglucosamine (GlcNAc), fucose, galactose, *N*-acetylneuraminic acid (Neu5Ac) and traces of mannose [32,33].

To date, the environmental factors and molecular mechanisms that drive the bacterium to colonize its reservoirs in the aquatic ecosystem remain largely unknown. In this study we found that, among the several oligosaccharides found in mucin, two components (Neu5Ac and GlcNAc) specifically trigger motility of *V*. *cholerae* O1. The catabolic pathways for Neu5Ac and GlcNAc converge, and the production of one catabolite that the two pathways share in common, glucosamine-6-phosphate (GlcN-6P), is essential to induce motility in the presence of either of these molecules. Interestingly, we found that mutants that are unable to produce the shared catabolite GlcN-6P show highly reduced motility towards mucin. Furthermore, we determined that the production of GlcN-6P is necessary to induce motility of *V*. *cholerae* O1 in the presence of environmental reservoirs such as crustaceans or cyanobacteria but not the mucus-rich gills of oysters, suggesting a complex set of responses involving the induction of motility in *V*. *cholerae* O1.

The specificity of the response of clinical *V*. *cholerae* in the presence of GlcNAc and Neu5Ac was surprising. Thus, we investigated whether that was a conserved and widespread mechanism in other members of the family Vibrionaceae. We compared the motility responses of environmental strains of *V*. *cholerae*, clinical and environmental isolates of *V*. *vulnificus* and *V*. *parahaemolyticus*, and fish and squid isolates of *V*. *fischeri* uncovering a wide variety of responses to different mucus oligosaccharides.

## Results

### Components of mucin trigger motility of *V*. *cholerae*

**Fig 1:**
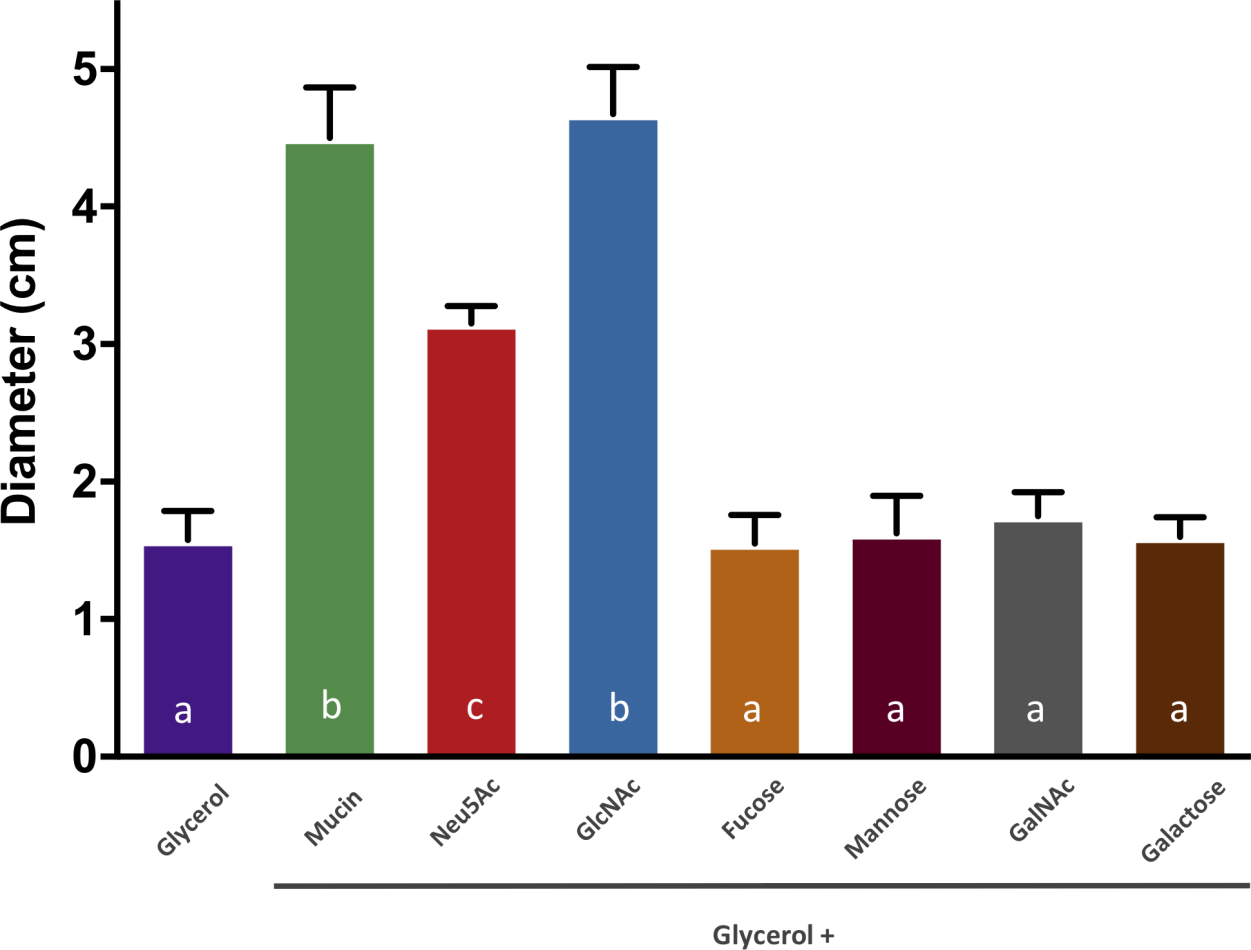
Motility of *V*. *cholerae* in the presence of mucus components. Motility assays of *V*. *cholerae* N16961 in glycerol supplemented with mucin and oligosaccharides found in mucin. The *y* axis denotes the diameter of the motility zone in cm and the *x* axis indicates the carbon source added to the motility plates. Columns represent the mean of four independent experiments and error bars the standard deviation. Statistical comparisons used one-way ANOVA followed by Tukey’s HSD test; bars with the letter a are not significantly different at alpha = 0.05 from one another but are different from b and c at P < 0.0001. Furthermore, b and c are also significantly different from each other with a P < 0.0001.

Mucins consist of a heavily glycosylated peptide backbone, with the glycosylated regions constituting up to 80% of the structure [32,33]. First, we tested the motility of the clinical O1 strain *V*. *cholerae* N16961 on a simple carbon source, glycerol, in M9 minimal media as a controlled proxy for baseline motility. We found that on soft agar M9 + minimal medium plates with 0.1% glycerol, *V*. *cholerae* shows an average diameter of 1.5 cm (Fig 1). Subsequently, we tested the influence of mucin on the motility of *V*. *cholerae* N16961, as it had been previously shown to induce the motility of *V*. *cholerae* (Fig 1) [25]. The diameter of the motility zone of *V*. *cholerae* N16961 wild-type in M9 minimal medium plates 0.1% glycerol supplemented with 0.01% mucin is approximately 4 cm. The CFU/ml between cultures grown for 24h in liquid M9 minimal medium supplemented with 0.1% glycerol or 0.1% glycerol + 0.01% mucin were not significantly different, suggesting that the difference in motility is not due to enhanced growth in the presence of mucin (S1 Fig). Rather, these results indicated that some components of mucin induced motility of *V*. *cholerae* N16961. Given their abundance in its composition, we tested whether the oligosaccharides commonly found in mucin induced motility of *V*. *cholerae* N16961. Supplementation of M9 + 0.1% glycerol plates with 0.01% Neu5Ac or GlcNAc greatly increased the motility of *V*. *cholerae* N16961 (Fig 1). The final concentration of oligosaccharides was below physiological level [32,33]. Again, the addition of these compounds had no effect on the number of CFU/ml when compared to M9 + 0.1% glycerol, suggesting that the increase in the measured motility zone was not due to excess carbon for growth (S1 Fig). Conversely, supplementation with 0.01% fucose, mannose, *N*-acetylgalactosamine (GalNAc), or galactose did not affect the motility of *V*. *cholerae* compared to the baseline on 0.1% glycerol plates, indicating that these compounds, which are the other oligosaccharides associated to mucin glycoproteins, do not trigger a chemotactic response in the bacterium (Fig 1). Overall, our results indicate that two of the several mucus oligosaccharides specifically induce an increase in the motility of *V*. *cholerae* N16961: Neu5Ac and GlcNAc.

### The catabolic pathways of Neu5Ac and GlcNAc converge

**Fig 2:**
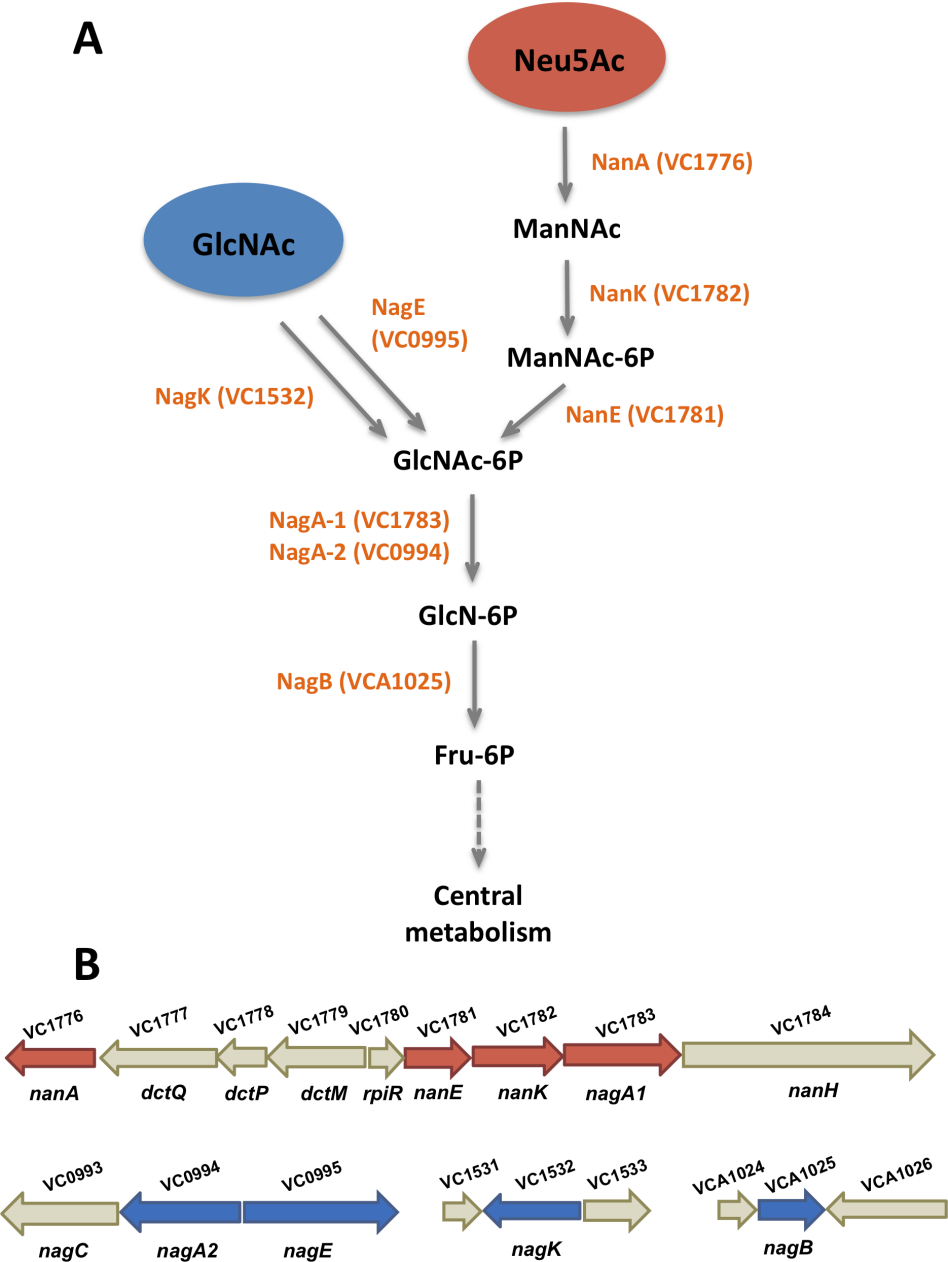
Schematic of Neu5Ac and GlcNAc catabolic pathways. A) The catabolic pathway of *N*-acetylneuraminic acid (Neu5Ac, red circle) converges with the catabolic pathway of *N*-acetylglucosamine (GlcNAc, blue circle) through the production of three common catabolites: *N*-acetylglucosamine-6-phosphate (GlcNAc-6P), glucosamine-6-phosphate (GlcN-6P) and fructose-6-phosphate (Fru-6P). Abbreviations are as follows; *N*-acetylmannosamine (ManNAc), *N*-acetylmannosamine-6-phosphate (ManNAc-6P), *N*-acetylglucosamine-6-phosphate (GlcNAc-6P). Names of enzymes and the locus tags of the genes that encode them are shown in orange and are abbreviated as follows; *N*-acetylglucosamine-specific IIA component (NagE), *N*-acetylglucosamine kinase, (NagK), *N*-acetylneuraminate lyase, (NanA), *N*-acetylmannosamine kinase (NanK), *N*-acetylmannosamine-6-phosphate epimerase (NanE), *N*-acetylglucosamine-6-phosphate deacetylase 1 (NagA-1) and 2 (NagA-2), glucosamine-6-phosphate deaminase (NagB). B) Arrow diagrams of Neu5Ac and GlcNAc catabolic clusters. Red and blue arrows represent genes encoding enzymes involved in the catabolism of Neu5Ac and GlcNAc. Grey arrows represent genes encoding genes with other functions such as Neu5Ac scavenging (*nanH*) and transport (*dctQ*, *dctP*, and *dctM*) or regulators (*nagC* and *rpiR*). Genes without a label underneath encode hypothetical proteins.

Utilization of the aminosugars GlcNAc and Neu5Ac as carbon sources has been previously reported to increase the fitness of *V*. *cholerae* in the mouse intestine [34,35]. Here, we examined the pathways involved in their catabolism (Fig 2). Exogenous GlcNAc is converted to *N*-acetylglucosamine-6-phosphate (GlcNAc-6P) by the *N*-acetylglucosamine-specific IIA component, NagE (VC0995), whereas endogenous GlcNAc is converted to GlcNAc-6P by *N*-acetylglucosamine kinase, NagK (VC1532) [34] (Fig 2). The genes involved in the catabolism of Neu5Ac are encoded within the Vibrio Pathogenicity Island-2, a mobile genetic element associated with pathogenic isolates of *V*. *cholerae* [35–38]. As shown in Fig 2, the catabolic pathway of Neu5Ac is initiated by its conversion into *N*-acetylmannosamine (ManNAc) by *N*-acetylneuraminate lyase, NanA (VC1776). *N*-acetylmannosamine kinase, NanK (VC1782), adds a phosphate group to ManNAc yielding *N*-acetylmannosamine-6-phosphate (ManNAc-6P) (Fig 2). Subsequently, *N*-acetylmannosamine-6-phosphate epimerase, NanE (VC1781), converts ManNAc-6P into GlcNAc-6P. Remarkably, the pathways of Neu5Ac and GlcNAc converge at this metabolic step, highlighting a link between the catabolism of the two aminosugars. According to KEGG pathway annotation of the genome of *V*. *cholerae* N16961, the breakdown of Neu5Ac and GlcNAc are the only two metabolic pathways leading to the production of GlcNAc-6P. *V*. *cholerae* encodes two enzymes involved in the catabolism of GlcNAc-6P into *N*-glucosamine-6-phosphate (GlcN-6P): *N*-acetylglucosamine-6-phosphate deacetylase 1, NagA-1, and 2, NagA-2 (VC0994 and VC1783 respectively) [34,35,39]. These enzymes appear to be exchangeable in order to catabolize GlcNAc-6P and their specific contributions to the catabolic pathway remain to be determined [34]. NagA-1 is encoded in the ancestral genome adjacent to NagE, whereas NagA-2 is encoded in VPI-2 in the cluster of genes involved in the catabolism of Neu5Ac (nan-nag cluster) [34,35]. GlcN-6P is further metabolized by glucosamine-6-phosphate deaminase, NagB (VCA1025), into Fructose-6-phosphate (Fru-6P), which enters central metabolism (Fig 2).

### Catabolism of Neu5Ac or GlcNAc is required to induce motility

We determined that Neu5Ac and GlcNAc induce motility of *V*. *cholerae* N16961 and their catabolic pathways converge. Our findings prompted us to study the molecular mechanisms behind the induction of motility and its possible relationship with the catabolism of these aminosugars. We constructed deletion mutants for the Neu5Ac pathway: Δ*nanA* and Δ*nanEK*. We decided to construct a double *nanE* and *nanK* mutant as the genes are poorly characterized in *V*. *cholerae* N16961 and it was previously shown that they overlap within an operon without clear origin and termination between them [35]. We also made deletions in the genes involved in the GlcNAc catabolic pathway: Δ*nagE* and Δ*nagK*, and the convergent part between the two pathways, Δ*nagA1*, Δ*nagA2*, Δ*nagA1-A2*, and Δ*nagB* (Fig 2). All these strains appear to have an intact flagellum under the microscope (data not shown).

**Fig 3:**
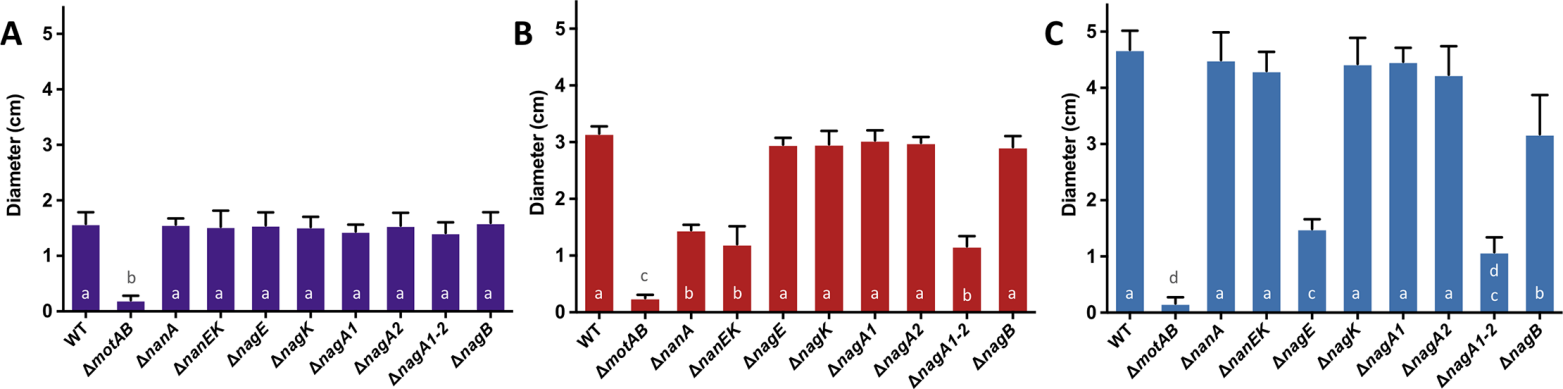
Motility of *V*. *cholerae* mutants in the presence of Neu5Ac and GlcNAc. The motility responses of different mutants involved in the catabolism of Neu5Ac and GlcNAc were tested on soft agar plates containing A) 0.1% glycerol or 0.1% glycerol supplemented with B) Neu5Ac and C) GlcNAc. Columns represent the mean of four independent experiments and error bars the standard deviation. Wild-type N16961 (WT), Δ*motAB* (non-motile), Δ*nanA* and Δ*nanEK* (cannot use Neu5Ac as a carbon source), Δ*nagE* and Δ*nagK* (cannot use exogenous or endogenous GlcNAc as a carbon source respectively), Δ*nagA1-A2* and Δ*nagB* (cannot use Neu5Ac or GlcNAc as carbon sources). Statistical comparisons were made using one-way ANOVA and comparing diameters of the mutants relative to wild-type using Tukey’s HSD test; bars within a panel with the same letter are not significantly different at alpha = 0.05. In A bars with the letter a are not significantly different at alpha = 0.05 from one another but are different from b at P < 0.0001. In B bars with the letter a are not significantly different at alpha = 0.05 from one another but are different from b and c at P < 0.0001. Furthermore, b and c are also significantly different from each other with a P < 0.0001. In C bars with the letter a are not significantly different at alpha = 0.05 from one another but are different from b, c and d at P < 0.0001. Furthermore, b and c are also significantly different from each other with a P < 0.0001.

We tested differential motility of these strains on soft agar plates containing M9 minimal media containing 0.1% glycerol as the sole carbon source (Fig 3A) and 0.1% glycerol supplemented with 0.01% Neu5Ac (Fig 3B) or 0.01% GlcNAc (Fig 3C). All strains, except for Δ*motAB*, a mutant lacking the flagellar motor protein genes used here as a negative control, showed similar baseline motility on 0.1% glycerol plates (Fig 3A). A mutant strain for the gene encoding the histidine kinase CheA2 (VC2063), which is involved in the chemotactic response of *V*. *cholerae*, was also tested for every condition [22]. The Δ*cheA2* mutant was not motile in any of the conditions tested (data not shown). On plates supplemented with Neu5Ac, the mutant strains Δ*nanA*, Δ*nanEK*, and Δ*nagA1-A2* showed similar motility to 0.1% glycerol plates, indicating that Neu5Ac must be catabolized in order to induce motility of *V*. *cholerae* N16961 (Fig 3B). Interestingly, a Δ*nagB* strain showed similar levels of motility as the wild-type strain suggesting that the NagB substrate (GlcN-6P) might be required for induction of motility whereas Fru-6P is not (Fig 2). On plates supplemented with GlcNAc, Δ*nagE* and Δ*nagA1-A2* mutants showed reduced motility, with a similar diameter to that of 0.1% glycerol plates, whereas Δ*nagK* showed a similar motility response as wild-type indicating that extracellular GlcNAc needs to be catabolized by NagE in order to promote motility of the bacterium (Fig 3C). Interestingly, Δ*nagB* mutant cells showed a slight decrease in motility compared to wild-type (Fig 3C). Overall, our data suggests that the production of the NagB substrate, GlcN-6P, is required for the drastic increase in motility of *V*. *cholerae* N16961 in the presence of both Neu5Ac and GlcNAc (Fig 3B and 3C).

In order to test whether exogenous addition of GlcN-6P or the non-phosphorilated GlcN could revert the wild-type phenotype of the Δ*nagA1-A2* mutant, we supplemented M9 minimal media soft agar plates with 0.1% glycerol plates with either 0.01% GlcN-6P or 0.01% GlcN. We found that neither the Δ*nagA1-A2* mutant nor the wild-type strain demonstrated an increase in motility compared to 0.1% glycerol plates (data not shown). To test whether *V*. *cholerae* N16961 encodes the transporter for GlcN-6P or GlcN we cultured wild-type *V*. *cholerae* in liquid M9 minimal medium supplemented with 0.1% GlcN-6P or 0.1% GlcN. No growth was detected after 24 hours on media containing 0.1% GlcN-6P, indicating that *V*. *cholerae* N16961 cannot take up exogenous GlcN-6P or use it as sole carbon source (data not shown). However, growth was detected after 24 hours on media containing 0.1% GlcN, indicating that *V*. *cholerae* N16961 can take up exogenous GlcN and use it as sole carbon source but it does not affect the motility of the bacterium (data not shown). Ectopic complementation of the Δ*nagA1-A2* mutant with an arabinose-inducible expression vector (pBAD22) encoding either *nagA1* or *nagA2* reverted wild-type motility levels in all substrates tested (data not shown). Overall, our results indicate that motility in the presence of Neu5Ac and GlcNAc is driven by the catabolism of these molecules. Furthermore, the intracellular production of GlcN-6P appears to be required in order to induce motility in the presence of GlcNAc or Neu5Ac.

### The inability to produce GlcN-6P leads to reduced motility in the presence of mucin

**Fig 4:**
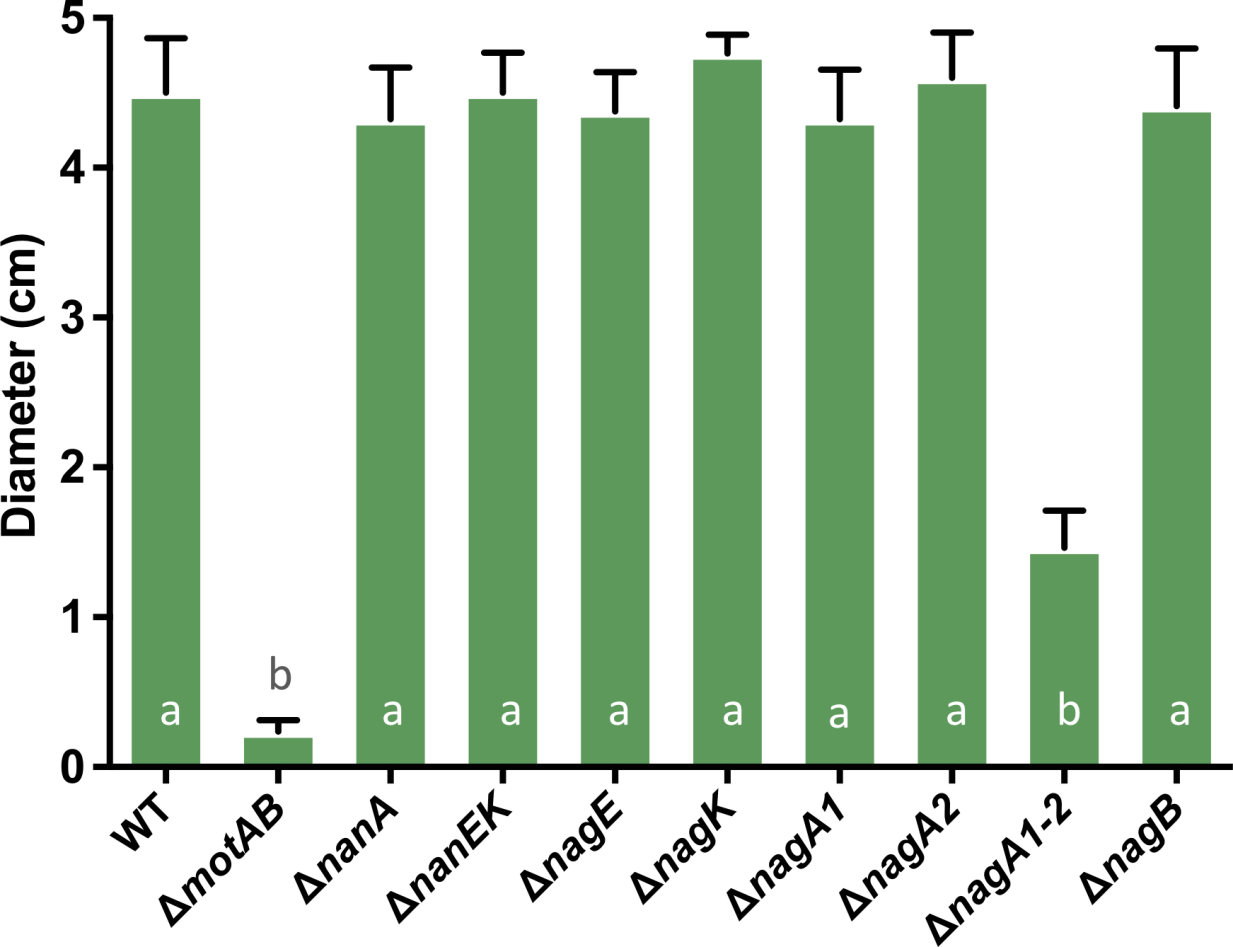
Motility *V*. *cholerae* mutants in the presence of mucin. The motility responses of different mutants involved in the catabolism of Neu5Ac and GlcNAc were tested on soft agar plates containing 0.1% glycerol supplemented with 0.01% mucin. Columns represent the mean of four independent experiments and error bars the standard deviation. Statistical comparisons were made using one-way ANOVA and comparing diameters of the mutants relative to wild-type using Tukey’s HSD test; bars within a panel with the same letter are not significantly different at alpha = 0.05. Bars with the letter a are not significantly different at alpha = 0.05 from one another but are different from b at P < 0.0001.

As described above, we determined that two of the aminosugars present in mucin triggered motility of *V*. *cholerae* N16961 and a common catabolite in their pathway, GlcN-6P, appears to be responsible for this increase in motility. We next studied the response of the different mutants in the presence of mucin (Fig 4). We tested each of the mutants on motility plates containing M9 + 0.1% glycerol supplemented with 0.01% mucin and found that the Δ*nagA1-A2* mutant showed significantly decreased motility on these plates (Fig 4), yet the number of CFU/ml after 24h in liquid media was similar to wild-type (S2 Fig). The Δ*cheA2* mutant was not motile in M9 + 0.1% glycerol supplemented with 0.01% mucin (data not shown). Interestingly, the Δ*nagB* mutant showed similar motility as the wild-type strain, suggesting that the production of GlcN-6P plays a crucial role in inducing motility of *V*. *cholerae* N16961 towards mucin.

### Catabolism of Neu5Ac and GlcNAc is involved in the motility of *V*. *cholerae* in the presence of environmental reservoirs

**Fig 5:**
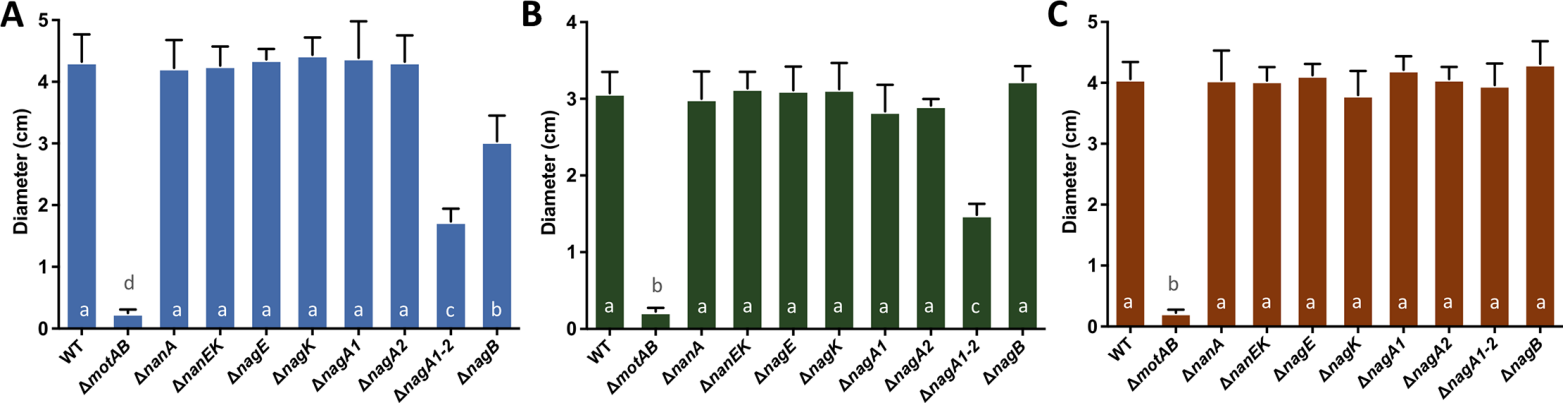
Motility of *V*. *cholerae* mutants in the presence of environmental reservoirs. The motility responses of different mutants involved in the catabolism of Neu5Ac and GlcNAc were tested on soft agar plates containing 0.1% Glycerol supplemented with 0.01% A) hexaacetyl-chitohexaose B) *Microcystis aeruginosa* C) oyster gill lysate. Columns represent the mean of four independent experiments and error bars the standard deviation. Wild-type N16961 (WT), Δ*motAB* (non-motile), Δ*nanA* and Δ*nanEK* (cannot use Neu5Ac as a carbon source), Δ*nagE* and Δ*nagK* (cannot use exogenous or endogenous GlcNAc as a carbon source respectively), Δ*nagA1-A2* (cannot use Neu5Ac or GlcNAc as carbon sources and is non-motile on mucin plates) and Δ*nagB* (cannot use Neu5Ac or GlcNAc as carbon sources). Statistical comparisons were made using one-way ANOVA and comparing diameters of the mutants relative to wild-type using Tukey’s HSD test; bars within a panel with the same letter are not significantly different at alpha = 0.05. In A bars with the same letter are not significantly different at alpha = 0.05 from one another. c and d are significantly different from a and b at P < 0.0001. In B and C bars with the letter a are not significantly different at alpha = 0.05 from one another but are different from b at P < 0.0001.

We next determined whether the aminosugars that induced motility of *V*. *cholerae* N16961 in the presence of mucin also played a role in the motility of the bacterium towards three prominent environmental reservoirs. GlcNAc and/or Neu5Ac are present in the chitinaceous surface of crustaceans, the sheath of cyanobacteria, and the mucus-rich gills of oysters [6,8]. We first tested the motility of the mutants in the presence of a soluble oligomer of chitin, hexaacetyl-chitohexaose, which makes up the shell of crustaceans. We found that all mutants, with the exception of Δ*motAB* and Δ*nagA1-A2*, were motile in soft agar plates of M9 minimal medium supplemented with 0.1% glycerol supplemented with 0.01% hexaacetyl-chitohexaose (Fig 5A). We also tested the motility of *V*. *cholerae* N16961 towards *Microcystis aeruginosa*, a cyanobacterium with which *V*. *cholerae* associates [11]. We found that the wild-type strain was highly motile in soft agar plates of M9 minimal medium supplemented with 0.1% glycerol supplemented with 0.01% *M*. *aeruginosa* (Fig 5B). All the mutants with the exception of Δ*motAB* and Δ*nagA1-A2* were motile on these plates, indicating that the production of GlcN-6P might also be required to trigger motility of *V*. *cholerae* N16961 towards this photosynthetic species. Finally, we tested the motility of *V*. *cholerae* N16961 in the presence of the homogenates of oyster gills as the bacterium has been found associated with this mucus-rich niche [40–43]. We found that all the strains we tested were highly motile in soft agar plates of M9 minimal medium containing 0.1% glycerol and supplemented with 0.01% oyster gills (Fig 5C). The Δ*cheA2* mutant was not motile in any of the conditions tested (data not shown). Our results indicate that the production of GlcN-6P plays a role in the motility of *V*. *cholerae* towards some of its environmental reservoirs (crustaceans and cyanobacteria) but not to others (oysters).

### Motility is differentially induced in environmental strains of *V*. *cholerae* and other *Vibrio* species

**Fig 6:**
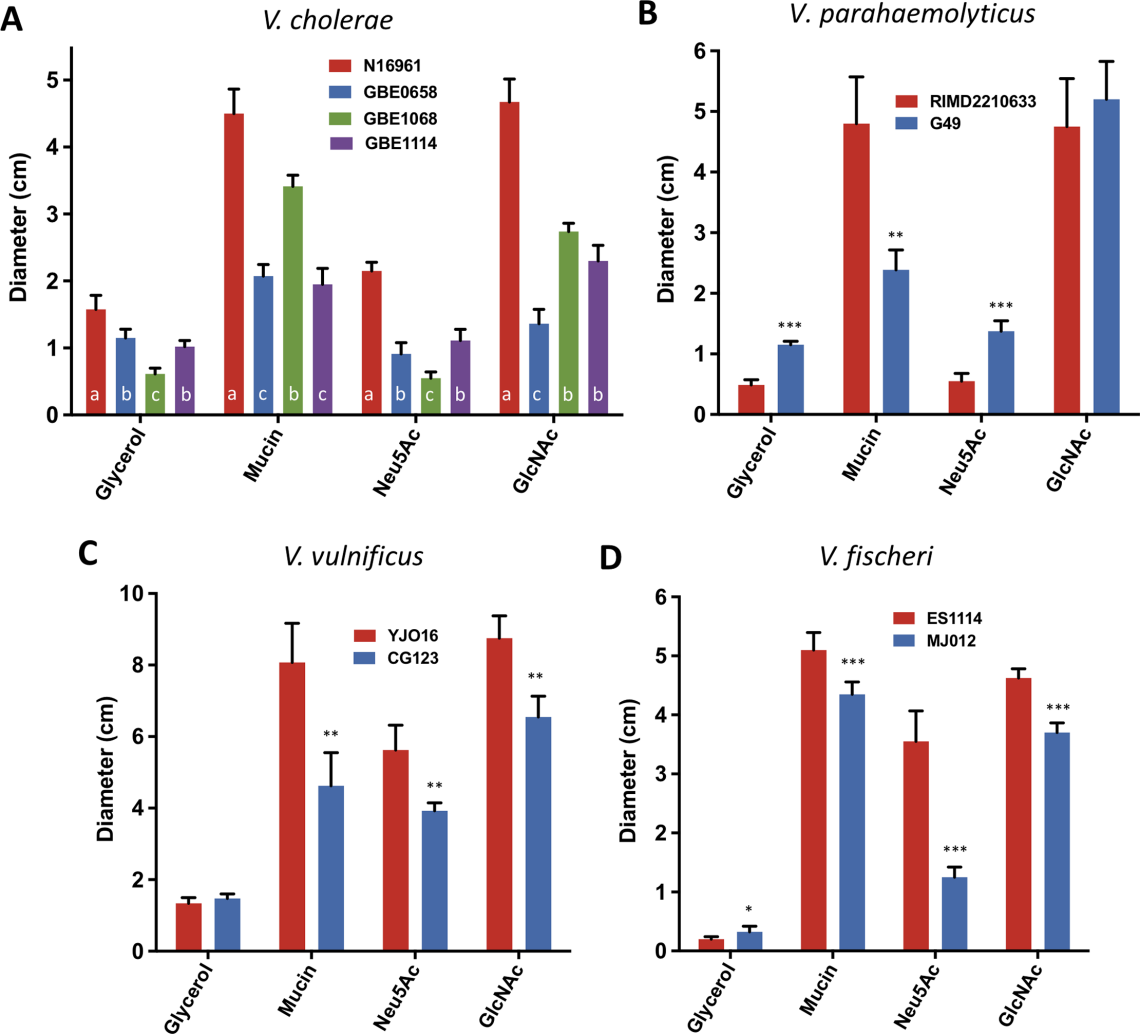
Motility of environmental strains of *V*. *cholerae* and other members of the family Vibrionaceae. The motility responses of: A) environmental strains of *V*. *cholerae* B) *V*. *parahaemolyticus* (red, clinical; blue, environmental) C) *V*. *vulnificus* (red, clinical; blue, environmental) D) *V*. *fischeri* (red, squid isolate; blue, fish isolate) were tested on soft agar plates containing glycerol, or glycerol supplemented with mucin, Neu5Ac or GlcNAc. Columns represent the mean of four independent experiments and error bars the standard deviation. Statistical comparisons in A were made using one-way ANOVA and comparing diameters of the environmental strains relative to N16961 using Tukey’s HSD test; bars within a panel with the same letter are not significantly different at alpha = 0.05. Bars with the letter a are significantly different from b and c at P < 0.0001. Furthermore, b and c are also significantly different from each other with a P < 0.0001. Statistical comparisons for B, C, and D were made using the student’s *t*-test and comparing diameters of environmental isolates relative to clinical isolates (B and C) or fish isolate relative to squid isolate (D). *P < 0.1, **P < 0.01, ***P < 0.001.

We found that a clinical strain of *V*. *cholerae* (N16961) showed very specific responses in the presence of different mucus components. We investigated whether that was a widespread behavior in other members of the family Vibrionaceae. First, we determined whether environmental strains of *V*. *cholerae* showed similar responses as the clinical one we tested. We studied the motility of three environmental strains isolated from the Great Bay Estuary of New Hampshire on M9 plates with 0.1% glycerol, and 0.1% glycerol plates supplemented with 0.01% mucin, Neu5Ac, or GlcNAc (Fig 6A) [42,44]. We found differences in motility among the environmental strains when compared to *V*. *cholerae* N16961, used here as control (Fig 6A). Mucin induced a moderate response in the environmental strains from the Great Bay Estuary GBE0658 and GBE1114, whereas another strain isolated in the same location, GBE1068, showed a significant increase in motility (Fig 6A). Interestingly, GBE1068 was isolated from oysters whereas GBE0658 and GBE1114 were isolated from water. GlcNAc was the only compound that we tested that induced a response in the environmental strain GBE1114. Only mucin triggered a response in the environmental strain GBE0658 (Fig 6A).

Lastly, we studied the chemotactic response towards the same compounds of interest in both clinical and environmental isolates of other members of the Vibrionaceae family: *V*. *parahaemolyticus* clinical (RIMD2210633) and environmental (G49) (Fig 6B), *V*. *vulnificus* clinical (YJO16) and environmental (CG123) (Fig 6C), *V*. *fischeri* fish (MJ012) and squid (ES1114) isolates (Fig 6D). We uncovered a wide variety of responses to the different compounds we tested. Generally, we found that clinical isolates showed a greater increase in motility in the presence of mucin than environmental isolates (Fig 6B and 6C). It is apparent that the responses in motility to different compounds varies widely between members of the Vibrionaceae and do not follow the response of *V*. *cholerae* O1.

## Discussion

In this study, we investigated the chemical triggers and molecular mechanisms that contribute to induce motility of *V*. *cholerae* O1 in the presence of some of its environmental reservoirs. Recently, Liu *et al* showed that the presence of mucin induces motility of *V*. *cholerae* and represses the synthesis of its polysaccharide [25]. Here, we identified two aminosugars found in mucin that promote movement of the bacterium: *N*-acetylneuraminic acid (Neu5Ac), and *N*-acetylglucosamine (GlcNAc) (Fig 1). It has been previously shown that both of these aminosugars can be utilized as a carbon and energy source by *V*. *cholerae* [13,34,35]. A reduced number of bacteria encode the genes required to catabolize Neu5Ac, a 9-carbon amino sugar commonly used to mediate cell-cell interactions and act as chemical messengers in eukaryotes [37]. GlcNAc, an 8-carbon aminosugar, is the main constituent of chitin, the most abundant polysaccharide in nature after cellulose [13]. Chitin is a ubiquitous source of carbon, nitrogen, and energy for marine microorganisms [13]. Here, we found that an intermediate catabolite of GlcNAc and Neu5Ac, GlcN-6P, triggers motility of *V*. *cholerae* in the presence of some of its common environmental reservoirs as well as mucin, thus acting potentially as an intracellular signaling molecule for motility. It appears that the bacterium has evolved convergent mechanisms to identify potential habitats to colonize in the environment and the site of infection in the human host, and to utilize the same carbon sources present in these distinct habitats.

The ability to use Neu5Ac and GlcNAc as carbon sources has previously been shown to confer a competitive advantage in the early stages of colonization of the mouse intestine [34,35]. Ghosh *et al*. showed that a Δ*nagA1-A2* mutant has a greatly reduced competitive index than a Δ*nagB* mutant (4-logs versus 2-logs, respectively) [34]. From the additional data presented here, it is possible that the competitive deficit seen in the Δ*nagA1-A2* mutant could be due to its inability to produce GlcN-6P, leading to reduced motility and subsequently lower colonization rates (Fig 4). Millet *et al*. recently showed that non-motile strains of *V*. *cholerae* are unable to successfully colonize the proximal and medial sections of the small intestine (SI) whereas they show only a slightly reduced fitness in the distal part of the SI [22]. Furthermore, they demonstrated that the abundance of mucin changes throughout the SI [22]. It is tempting to speculate that this difference might be due to the variation in the abundance and perhaps composition of mucins along the length of the SI, which would allow non-motile strains to reach the epithelium without the need of active swimming in the distal section of the SI. Interestingly, correct spatiotemporal regulation of GlcNAc utilization confers a fitness advantage to *V*. *fischeri* during colonization of the light organ of the Hawaiian squid *Euprymna scolopes* [45].

We investigated the motility of *V*. *cholerae* towards organisms that could serve as potential environmental sources of Neu5Ac or GlcNAc. We hypothesized a link between the colonization of the host intestine and known biotic substrates that are found in environmental reservoirs of *V*. *cholerae*. For example, pathogenic *V*. *cholerae* isolates have been found in association with the mucilaginous sheath of several cyanobacteria [9,10,46,47]. Moreover, *V*. *cholerae* frequently colonizes crustaceans, such as zooplankton and shellfish, whose chitinaceous exoskeletons are made of polymers of GlcNAc [30,48,49]. Surprisingly, the Δ*nagE* mutant was motile in the presence of hexaacetyl-chitohexaose, a soluble oligomer of chitin, indicating that *V*. *cholerae* might have alternative pathways for the uptake of GlcNAc polymers [13]. Furthermore, it appears that *V*. *cholerae* has developed alternate pathways to sense the presence of its diverse aquatic reservoirs as, unlike for chitin or *M*. *aeruginosa*, motility towards oysters appears to be independent from the production of GlcN-6P. It appears that biotic and abiotic factors in the natural environment of *V*. *cholerae* have driven the emergence of some of its virulence traits [44,50]. It is possible that the ability to recognize some of it potential reservoirs provided *V*. *cholerae* with preadaptations that confer an advantage during intestinal colonization.

Finally, we tested the motility responses of environmental isolates of *V*. *cholerae* and three other *Vibrio* species towards the mucin components that induce motility of clinical *V*. *cholerae*. From the subset of *V*. *cholerae* environmental strains that we studied we found that isolates from water and oysters differed in their motility responses, with the oyster isolate being the only environmental strain tested that showed a drastic increase in motility in the presence of mucin. One of the isolates we tested, GBE0658, did not show an increase in motility in the presence of any of the carbon sources. Overall, our data suggests that different *V*. *cholerae* isolates have a distinctive set of preferred motility inducers that might reflect the specific niches they colonize. We also tested the effect on motility that the same group of carbon sources had on several other *Vibrio* species. Interestingly, neither the environmental strains of *V*. *cholerae* nor the two *V*. *parahaemolyticus* strains that we tested encode the genes for sialic acid catabolism or showed an induction of motility in the presence of Neu5Ac (Fig 6). This suggests that a similar molecular mechanism that requires catabolism of a compound to induce motility might be widespread among the Vibrionaceae. Previous studies have demonstrated chemotaxis in *V*. *fischeri* towards GlcNAc and (GlcNAc)_2_, and *Vibrio anguillarum* is highly motile in the presence of the intestinal mucus of the trout [51,52]. It remains to be determined whether the presence of single or multiple polar flagella play a role in the differences in motility that we found as the Vibrio species we tested are known to be differently flagellated [53–55]. Our results highlight the vast intra and interspecies diversity in the responses that members of the family Vibrionaceae have towards nutrient sources.

Overall, our study sheds light on some of the environmental signals and molecular mechanisms that might drive environmental colonization of *V*. *cholerae*. Further knowledge is required to better understand how the bacterium persists in the aquatic environment and how this affects its ability to infect humans and cause disease.

## Material and methods

### Bacterial strains and plasmids

*V*. *cholerae* N16961 was used as the wild-type strain. Environmental *V*. *cholerae*, *V*. *fischeri* and *V*. *parahaemolyticus* strains were kindly provided by Dr. Cheryl Whistler. *V*. *vulnificus* strains were kindly provided by Dr. E. Fidelma Boyd. Strains cultivated on solid medium were grown on 1.5% LB agar; strains in liquid media were grown in aerated LB broth at 37°C, with the exception of *V*. *fischeri*, which was cultivated on LBS medium at 28°C. pKAS154 was used for allelic exchange. pBAD22 was used as expression vector. When necessary, media was supplemented with antibiotics to select for certain plasmids or *V*. *cholerae* at the following concentrations: kanamycin, 45 μg/ml; polymyxin B, 50 units/ml; ampicillin, 30 μg/ml and streptomycin, 1 mg/ml. In order to induce expression of genes cloned in pBAD22 100μg L-Arabinose was added per ml of culture.

### Strain construction and visualization

In-frame deletions of genes of interest were constructed via homologous recombination. PCR was used to amplify two approximately 500 bp fragments flanking the gene and to introduce restriction sites. The fragments were then cloned into a restriction-digested plasmid using a three-segment ligation. The resulting plasmid was electroporated into *Escherichia coli* S17-1λpir. *E*. *coli* with the constructed plasmid was mated with *V*. *cholerae* N16961, and allelic exchange was carried out by selection on antibiotics, as described previously [56]. Potential mutants were screened using PCR: two primers flanking the deletion construct were used to amplify chromosomal DNA isolated from plated *V*. *cholerae*. The lengths of the PCR fragments were analyzed on 0.8% agarose for gene deletions and putative deletions were subsequently confirmed by DNA sequencing. Strains were inspected for presence of intact flagellum using a Carl Zeiss Axio Observer 7 Advanced Motorized Inverted Microscope.

### Motility assays

Motility in the presence of different compounds was quantified by measuring the diameter of one colony after 24 hours of growth at 37°C on a semi-solid (0.3%) agar plate. MSR media at 28°C was used for *V*. *fischeri*. M9 minimal medium + 0.1% glycerol was used to measure baseline motility. 0.1% glycerol plates were supplemented either with 0.01% porcine mucin, 0.01% *N*-acetylglucosamine (GlcNAc), *N*-acetylneuraminic acid (Neu5Ac), L-fucose, D-mannose, D-galactose, or *N*-acetylgalactosamine (GalNAc) to measure motility in the presence of these compounds (Sigma). Motility was also evaluated on soft agar plates containing M9 minimal medium and 0.01% hexaacetyl-chitohexaose (Megazyme). *Microcystis aeruginosa* was grown on MBL medium at 22°C in the presence of full light with light shaking to avoid flocculation. *M*. *aeruginosa* was added 0.01% w/v. The mucus-containing gills of *Crassostrea spp*. were homogenized and autoclaved. *Crassostrea spp*. gill homogenate was added to 0.01% w/v. Results were plotted using Prism software and the statistical significance was obtained by using student’s *t*-test.

### CFU count

Cultures were inoculated from colonies grown overnight on LB medium plates at 37°C. 1:50 of overnight cultures were added to liquid M9 minimal medium containing 0.1% glycerol only or supplemented with either 0.01% porcine mucin, *N*-acetylglucosamine (GlcNAc), *N*-acetylneuraminic acid (Neu5Ac), L-fucose, D-mannose, D-galactose, *N*-acetylgalactosamine (GalNAc), glucosamine-6-phosphate (GlcN-6P) or glucosamine (GlcN). The cultures were incubated at 37°C for 24 hours in a rotary shaker. Serial dilutions of the cultures were plated on LB to calculate the number of CFU/ml of culture at that time point.

## Supporting information

S1 Fig Growth of *V*. *cholerae* in the presence of mucus components. CFU/ml of *V*. *cholerae* N16961 in liquid M9 minimal media with glycerol or glycerol supplemented with mucin and oligosaccharides found in mucin. The *y* axis denotes the CFU/ml and the *x* axis indicates the carbon source added to the media. Columns represent the mean of three independent experiments and error bars the standard deviation. Statistical comparisons were made using the student’s *t*-test and comparing samples relative to 0.1% glycerol.
(DOCX)

S2 Fig Growth of *V*. *cholerae* mutants in M9 minimal media with glycerol supplemented with mucin. The *y* axis denotes the CFU/ml and the *x* axis indicates the mutant that was tested. Columns represent the mean of three independent experiments and error bars the standard deviation. Statistical comparisons were made using the student’s *t*-test and comparing the mutants relative to WT. Wild-type N16961 (WT), Δ*motAB* (non-motile), Δ*nagA1-A2* (cannot use Neu5Ac or GlcNAc as carbon sources and is non-motile on mucin plates) and Δ*nagB* (cannot use Neu5Ac or GlcNAc as carbon sources).
(DOCX)
