# Supplementary material for: Catabolism of mucus components influences motility of Vibrio cholerae in the presence of environmental reservoirs
Source: PLoS One. 2018 Jul 26;13(7):e0201383. doi: 10.1371/journal.pone.0201383 (PMC6062102; doi:10.1371/journal.pone.0201383)
Supplement: S1 Fig — CFU/ml of V. cholerae N16961 in liquid M9 minimal media with glycerol or glycerol supplemented with mucin and oligosaccharides found in mucin. The y axis denotes the CFU/ml and the x axis indicates the carbon source added to the media. Columns represent the mean of three independent experiments and error bars the standard deviation. Statistical comparisons were made using the student’s t-test and comparing samples relative to 0.1% glycerol. (DOCX) [file pone.0201383.s001.docx]

**S1 Fig. Growth of *V. cholerae* in the presence of mucus components.** CFU/ml of *V. cholerae* N16961 in liquid M9 minimal media with glycerol or glycerol supplemented with mucin and oligosaccharides found in mucin. The *y* axis denotes the CFU/ml and the *x* axis indicates the carbon source added to the media. Columns represent the mean of three independent experiments and error bars the standard deviation. Statistical comparisons were made using the student’s *t*-test and comparing samples relative to 0.1% glycerol.
