# Supplementary material for: Catabolism of mucus components influences motility of Vibrio cholerae in the presence of environmental reservoirs
Source: PLoS One. 2018 Jul 26;13(7):e0201383. doi: 10.1371/journal.pone.0201383 (PMC6062102; doi:10.1371/journal.pone.0201383)
Supplement: S2 Fig — The y axis denotes the CFU/ml and the x axis indicates the mutant that was tested. Columns represent the mean of three independent experiments and error bars the standard deviation. Statistical comparisons were made using the student’s t-test and comparing the mutants relative to WT. Wild-type N16961 (WT), ΔmotAB (non-motile), ΔnagA1-A2 (cannot use Neu5Ac or GlcNAc as carbon sources and is non-motile on mucin plates) and ΔnagB (cannot use Neu5Ac or GlcNAc as carbon sources). (DOCX) [file pone.0201383.s002.docx]

**S2 Fig. Growth of *V. cholerae* mutants in M9 minimal media with glycerol supplemented with mucin.** The *y* axis denotes the CFU/ml and the *x* axis indicates the mutant that was tested. Columns represent the mean of three independent experiments and error bars the standard deviation. Statistical comparisons were made using the student’s *t*-test and comparing the mutants relative to WT. Wild-type N16961 (WT), ∆*motAB* (non-motile), ∆*nagA1-A2* (cannot use Neu5Ac or GlcNAc as carbon sources and is non-motile on mucin plates) and ∆*nagB* (cannot use Neu5Ac or GlcNAc as carbon sources)
